# Factors associated with *de novo* urolithiasis after kidney transplantation: a matched case-control study

**DOI:** 10.3389/fmed.2026.1866555

**Published:** 2026-09-21

**Authors:** Wenshi Tian, Zhengyang Zhang, Hao Chen, Xulei Wang, He Jiang, Kai Liu, Dongdong Chen, Bin Shen, Jiwei Yang, Xianduo Li, Xiaoming Zhang, Yunchao Wang, Jianning Wang

**Affiliations:** 1 Shandong Provincial Qianfoshan Hospital, Shandong University, Jinan, China; 2 Department of Urology, The First Affiliated Hospital of Shandong First Medical University & Shandong Provincial Qianfoshan Hospital, Shandong Institute of Nephrology, Jinan, China

**Keywords:** *de novo* urolithiasis, kidney transplantation, risk-set matching, serum phosphate, serum urate

## Abstract

**Objective:**

To investigate the clinical characteristics and potential risk factors for *de novo* urolithiasis in recipients after kidney transplantation.

**Methods:**

We retrospectively screened a source cohort of 2,301 kidney transplant recipients undergoing longitudinal follow-up between January 2015 and December 2025. Forty recipients who developed *de novo* post-transplant urolithiasis were identified. For each recipient who developed a stone, the date of first stone diagnosis was defined as the index date. Controls who had undergone transplantation and remained under follow-up without detected stones at the corresponding index date were selected using chronological risk-set matching at a maximum 1:2 ratio. Thirty-seven cases were matched to 74 controls. Laboratory exposures were summarized using available baseline/pre-index measurements. Covariate balance was assessed using standardized mean differences (SMDs). The primary analysis used conditional logistic regression, with Firth bias-reduced conditional logistic regression as a sensitivity analysis.

**Results:**

Among 2,301 recipients, 40 developed *de novo* post-transplant urolithiasis, corresponding to an observed cumulative proportion of 1.74%. The median time from transplantation to first stone diagnosis was 341.5 days (interquartile range, 41.0–1,931.5 days). After risk-set matching, excellent covariate balance was achieved across all matched variables (maximum absolute SMD = 0.037). Standard conditional logistic regression revealed that higher baseline serum urate was significantly associated with an increased risk of *de novo* urolithiasis [odds ratio (OR) 1.72 per 50 μmol/L increase, 95% CI 1.17–2.54; P = 0.006], whereas higher serum phosphate exhibited an inverse association (OR 0.77 per 0.1 mmol/L increase, 95% CI 0.62–0.95; *P* = 0.016). Firth sensitivity analysis confirmed these findings for serum urate (OR 1.64, 95% CI 1.13–2.38; *P* = 0.009) and serum phosphate (OR 0.79, 95% CI 0.65–0.97; *P* = 0.025). Urinary white blood cell (WBC) count showed a strong exploratory association (OR 2.71 per log2 doubling, 95% CI 1.59–4.63; *P* < 0.001), though the model exhibited near-separation.

**Conclusions:**

Elevated serum urate is consistently associated with an increased risk of *de novo* urolithiasis following kidney transplantation. Lower serum phosphate represents a potential secondary metabolic signal requiring further validation. While urinary leukocyturia strongly correlated with stone status in exploratory models, it should be interpreted cautiously and not regarded as a definitive causal factor or surrogate for culture-confirmed urinary tract infection.

## Introduction

Kidney transplantation remains the gold standard of care for patients with end-stage renal disease (ESRD) (1). With the maturation of surgical techniques and the optimization of immunosuppressive regimens, recipient survival has extended significantly, and long-term post-transplant complications have garnered increasing clinical attention. *de novo* urolithiasis following kidney transplantation is a relatively uncommon but potentially severe complication, with systematic reviews reporting an incidence rate between 0.4% and 4.4% (2). Compared to the general population, the pathophysiological mechanisms of stone formation in transplant recipients are more complex due to altered anatomical structures (e.g., ureteroneocystostomy), chronic immunosuppression, and metabolic derangements such as hyperuricemia and hyperoxaluria.

The clinical presentation of stones in the allograft is often atypical. Because the transplanted kidney is denervated, the classic symptoms of renal colic are frequently absent, often leading to delayed diagnosis (3). If left untreated, urolithiasis can result in urinary tract obstruction, recurrent infections, and even acute kidney injury or total graft loss. Therefore, early identification of risk factors and the implementation of effective preventive and therapeutic measures are of paramount importance.

However, factors driving post-transplant urolithiasis remain insufficiently defined. Existing studies are limited by small sample sizes, heterogeneous study populations, or failure to account for unequal post-transplant follow-up duration (4, 5). Current stone guidelines recommend stone analysis and metabolic evaluation, including assessment of serum creatinine, calcium, phosphate, uric acid, urine pH, and urine culture when infection is suspected (6). To address previous methodological limitations, this study evaluated the clinical characteristics and metabolic predictors of *de novo* allograft urolithiasis using chronological risk-set matching combined with matched conditional logistic regression.

## Materials and methods

### Study population

This was a single-center retrospective observational study conducted at the First Affiliated Hospital of Shandong First Medical University. The source cohort comprised 2,301 kidney transplant recipients under longitudinal follow-up during the stone-ascertainment period from January 2015 to December 2025. *De novo* urolithiasis was defined as a newly detected urinary stone after transplantation, excluding stones known to be present in the donor kidney at procurement.

The exclusion criteria were as follows: (a) donor-derived urolithiasis, defined as stones present in the donor kidney at procurement; (b) inadequate longitudinal records to determine stone status during the relevant observation period; and (c) insufficient baseline or matching information for the matched analysis. Recipients with missing matching covariates or an unparseable follow-up endpoint were excluded from the risk-set matching pool. No statistical imputation was performed.

The study was approved by the Ethics Committee of the First Affiliated Hospital of Shandong First Medical University (approval number: YXLL-KY-2025[029]). All participants provided written informed consent.

### Risk-set matching and matched cohort construction

To mitigate baseline confounding and control for unequal time-at-risk, chronological risk-set matching was implemented. For each index case, the index date was assigned as the date of initial post-transplant stone diagnosis. Eligible controls were defined as recipients who had undergone transplantation prior to the index date, remained under active follow-up, and were free of diagnosed urolithiasis at or before that index date.

Exact matching was performed for biological sex and pre-transplant history of urolithiasis. Continuous matching variables—age, height, weight, BMI, presence of hypertension, and diabetes mellitus—were standardized using overall population standard deviations and combined using Euclidean nearest-neighbor distance. Matching was performed chronologically by index date at a maximum 1:2 ratio without replacement. Out of 2,260 candidate controls, 2,033 possessed complete time and covariate datasets. Ultimately, 37 stone cases were successfully matched to 74 unique controls. Three unmatched cases were retained solely for descriptive reporting. Post-matching covariate balance was verified via Standardized Mean Differences (SMDs), with |SMD|<0.10 defined as optimal balance (Figure 1).

**Figure 1:**
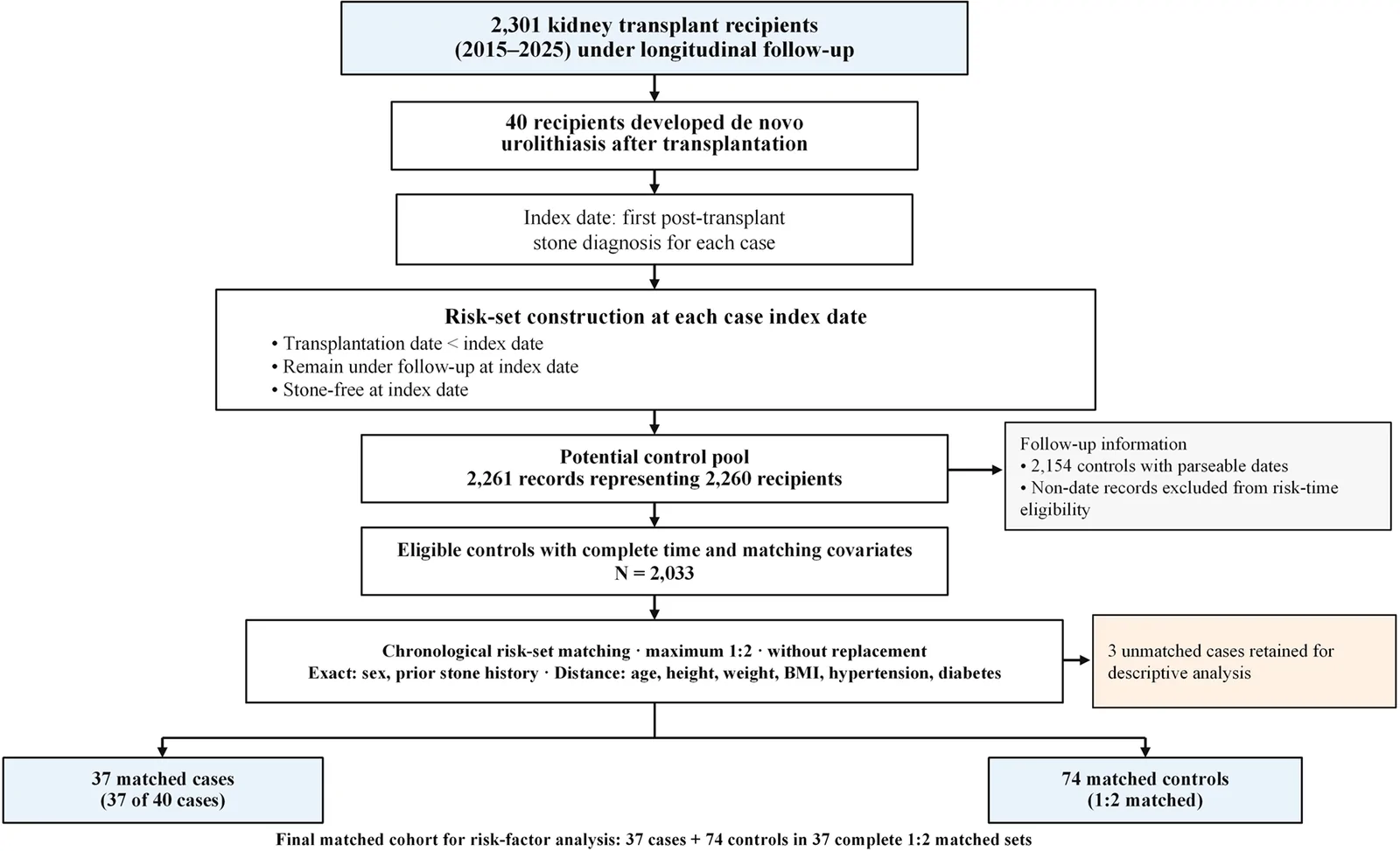
Flow diagram of cohort construction and 1:2 chronological risk-set matching. Of the 40 recipients who developed *de novo* urolithiasis, 37 were matched to 74 unique stone-free controls, forming 37 matched sets. Three unmatched cases were retained for descriptive analyses only.

### Variable definitions and clinical data collection

Baseline variables included recipient demographics, comorbidity status, donor type, graft function metrics, and metabolic/urinalysis profiles. For matched controls, laboratory parameters were extracted from clinical records obtained prior to or on the corresponding index date. To minimize transient intra-individual fluctuations, serum urate, serum calcium, urine pH, urinary white blood cell (WBC) count, and urinary bacterial count were summarized as the average of up to three consecutive measurements obtained prior to the index date. Serum phosphate was analyzed using the baseline average. Serum urate was evaluated as a continuous metric. Because culture-confirmed urinary tract infection (UTI) data were not uniformly collected across the historical cohort, urinary WBC count was analyzed as a continuous urinalysis marker rather than a surrogate for culture-proven UTI.

### Statistical analysis

Statistical analyses were performed using SPSS Statistics (version 26.0; IBM Corp., Armonk, NY, USA) and Python 3.13.5 (SciPy 1.17.0 and statsmodels 0.14.6). Distributional assumptions were evaluated using histograms, Q–Q plots, and the Shapiro–Wilk test. Approximately normally distributed continuous variables are summarized as mean ± standard deviation, whereas skewed variables are summarized as median (IQR). Covariate balance before and after matching was evaluated using SMDs rather than hypothesis-testing *P*-values. The primary matched analysis used conditional logistic regression stratified by matched set. To limit overfitting, the primary metabolic/renal-function model included four clinically relevant variables with complete matched-set data: serum urate (per 50 μmol/L), serum phosphate (per 0.1 mmol/L), serum calcium (per 0.1 mmol/L), and serum creatinine (per 10 μmol/L), yielding 37 events for four parameters (events per variable = 9.25). Firth bias-reduced conditional logistic regression, implemented using a Jeffreys-prior penalty, was performed as the principal sensitivity analysis to reduce small-sample and separation bias (7). Urinary WBC counts were markedly right-skewed and transformed as log2(WBC+1); because of potential reverse causation and near-separation, urinary WBC was analyzed as an exploratory matched factor. Missing observations were handled by complete-case analysis without imputation. All tests were two-sided, and *P* < 0.05 was considered statistically significant.

## Results

### Incidence and baseline characteristics

Within the observation period, 40 of 2,301 recipients developed *de novo* allograft urolithiasis (observed cumulative proportion: 1.74%). Among the 40 cases, 33 (82.5%) were male, with a mean age of 43.23 ± 12.01 years. The median time from transplantation to initial stone diagnosis was 341.5 days (IQR: 41.0–1,931.5 days; range: 27–5,286 days). Nineteen cases (47.5%) were diagnosed within 6 months, and 21 (52.5%) were diagnosed within 1 year post-transplant. Figure 2 summarizes recipient demographics, stone characteristics, and the timeline of diagnosis.

**Figure 2:**
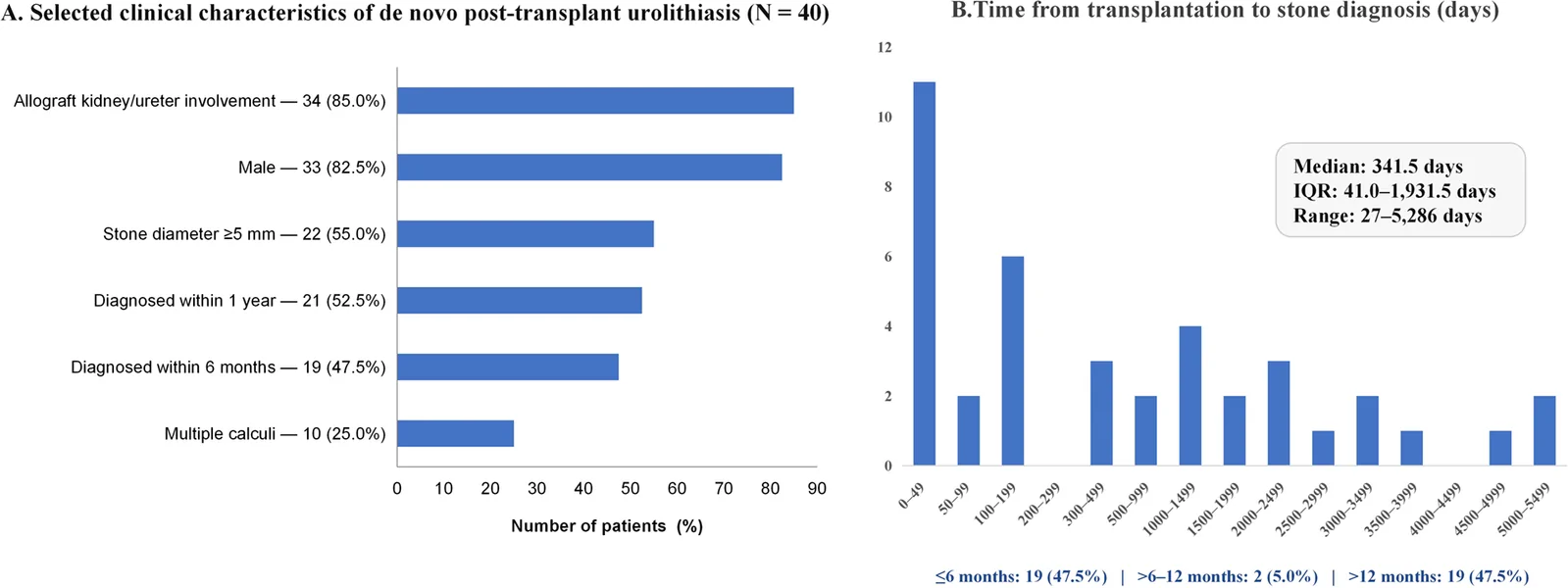
Clinical characteristics and timing of *de novo* urolithiasis after kidney transplantation. **(A)** Selected clinical characteristics and stone features among the 40 recipients who developed *de novo* post-transplant urolithiasis. **(B)** Distribution of time from kidney transplantation to the first diagnosis of *de novo* urolithiasis. Time intervals were 0–49, 50–99, 100–199, 200–299, 300–499 days, followed by 500-day intervals. The median time to stone diagnosis was 341.5 days (IQR, 41.0–1,931.5 days; range, 27–5,286 days).

### Risk-set matching and balance assessment

The 1:2 risk-set matching procedure yielded 37 matched case-control sets (37 cases and 74 unique controls). Three cases could not be matched under strict distance thresholds and were retained only for descriptive analysis. Prior to matching, substantial baseline imbalances existed between cases and candidate controls in weight (|SMD| = 0.341), prior stone history (0.287), BMI (0.249), and height (0.246). Following matching, all covariates demonstrated excellent balance with |SMD|<0.10 (maximum post-match |SMD| of 0.037, Table 1; Supplementary Figure S1).

**Table 1:** Covariate balance before and after risk-set matching. SMD, standardized mean difference. Balance was considered acceptable when |SMD|<0.10.

Covariate	All cases (*n* = 40)	Eligible controls	Matched cases (*n* = 37)	Matched controls (*n* = 74)	SMD before	SMD after
Male sex	0.825	0.756	0.811	0.811	0.170	0.000
Age, years	43.225	43.414	43.892	44.284	−0.016	−0.033
Height, cm	172.025	170.142	172.189	172.216	0.246	−0.004
Weight, kg	71.035	66.640	71.416	71.000	0.341	0.037
BMI, kg/m²	23.966	22.959	24.059	23.937	0.249	0.035
Hypertension	0.900	0.897	0.892	0.892	0.011	0.000
Diabetes	0.175	0.117	0.189	0.189	0.165	0.000
Prior stone history	0.050	0.004	0.027	0.027	0.287	0.000

### Clinical outcomes and interventions

Imaging confirmed that 34 cases (85.0%) had stones located within the allograft parenchyma, renal pelvis, or transplant ureter. Therapeutic strategies were tailored individually based on stone burden, location, obstruction severity, graft anatomy, and clinical symptoms, including flexible ureteroscopy (fURS), extracorporeal shock-wave lithotripsy (ESWL), percutaneous nephrolithotomy (PCNL), ureteral stent management, or conservative observation. No complete graft failures directly attributable to stone obstruction or procedural complications occurred during follow-up.

### Matched multivariable association analyses

In the 37 matched case sets, the primary standard conditional logistic model included serum urate, phosphate, calcium, and creatinine. Higher serum urate was associated with *de novo* urolithiasis (OR 1.72 per 50 μmol/L, 95% CI 1.17–2.54; *P* = 0.006), whereas serum phosphate showed an inverse association (OR 0.77 per 0.1 mmol/L, 95% CI 0.62–0.95; *P* = 0.016). Serum calcium and creatinine were not statistically significant. Firth bias-reduced conditional logistic regression produced similar estimates: serum urate OR 1.64 (95% CI 1.13–2.38; *P* = 0.009), serum phosphate OR 0.79 (95% CI 0.65–0.97; *P* = 0.025), serum calcium OR 0.82 (95% CI 0.60–1.11; *P* = 0.191), and serum creatinine OR 0.97 per 10 μmol/L (95% CI 0.91–1.04; *P* = 0.426) (Table 2; Figure 3). The primary model included 37 events and four parameters (events per variable = 9.25), the design matrix was full rank, and no matched set had a fitted case probability >0.95 in the standard conditional model. Urinary WBC count was markedly skewed [median [IQR], 83.3 [25.8–185.0] cells/*μ*L in matched cases versus 3.8 [2.0–7.2] cells/μL in controls] and showed a strong exploratory Firth conditional association after log2(WBC+1) transformation (OR 2.71 per doubling, 95% CI 1.59–4.63; *P* < 0.001). The WBC model showed near-separation (maximum fitted case probability 0.999; 18/37 sets >0.95) and was interpreted cautiously. Culture-defined UTI was not separately analyzed.

**Table 2:** Matched conditional logistic and firth bias-reduced conditional logistic analyses.

Variable	Standard conditional OR (95% CI)	P	Firth conditional OR (95% CI)	P
Serum urate, per 50 μmol/L	1.72 (1.17–2.54)	0.006	1.64 (1.13–2.38)	0.009
Serum phosphate, per 0.1 mmol/L	0.77 (0.62–0.95)	0.016	0.79 (0.65–0.97)	0.025
Serum calcium, per 0.1 mmol/L	0.79 (0.57–1.08)	0.139	0.82 (0.60–1.11)	0.191
Serum creatinine, per 10 μmol/L	0.96 (0.90–1.04)	0.326	0.97 (0.91–1.04)	0.426
Urinary WBC, log2(mean+1)*	3.10 (1.63–5.91)	<0.001	2.71 (1.59–4.63)	<0.001

*Urinary WBC was analyzed separately as an exploratory model because of potential reverse causation and near-separation; it was not included in the primary four-variable metabolic/renal-function model.

**Figure 3:**
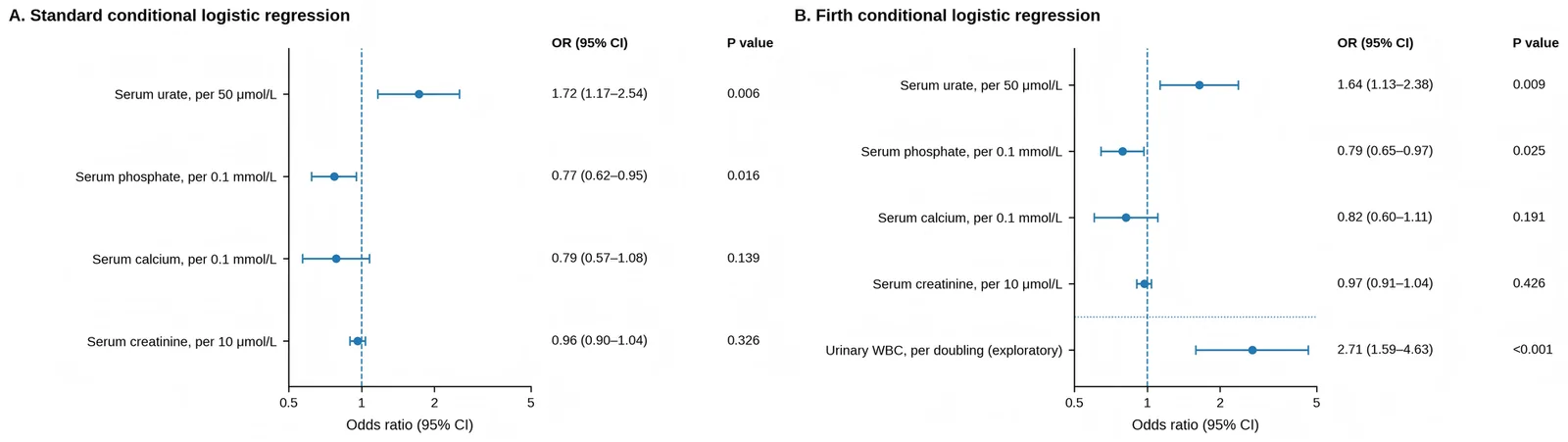
Forest plots of conditional regression analyses for factors associated with *de novo* urolithiasis after kidney transplantation in the 37 matched risk sets. **(A)** Standard conditional logistic regression. **(B)** Firth bias-reduced conditional logistic regression as a sensitivity analysis. Serum urate, serum phosphate, serum calcium, and serum creatinine were included in the primary multivariable model. Urinary white blood cell (WBC) count was evaluated separately as an exploratory factor because of potential reverse causation and sparse-data/near-separation concerns.

## Discussion

In this single-center cohort, *de novo* urolithiasis was identified in 40 of 2,301 kidney transplant recipients, corresponding to an observed cumulative proportion of 1.74%. The median time from transplantation to the first stone diagnosis was approximately 11 months. Chronological risk-set matching ensured that controls remained stone-free and under follow-up at each case index date and achieved excellent balance across the prespecified matching covariates. In matched analyses, higher serum urate was consistently associated with stone occurrence, whereas serum phosphate showed an inverse association. Serum calcium and creatinine were not independently associated. Similar estimates from Firth bias-reduced conditional logistic regression supported the robustness of these findings.

The link between hyperuricemia and post-transplant urolithiasis is clinically logical. Serum urate elevation in transplant recipients often stems from impaired renal excretion, calcineurin inhibitor (CNI) use, altered metabolic status, or graft dysfunction. While elevated serum urate predicts stone formation, it should not be assumed that all observed stones are composed of pure uric acid. Nonetheless, these findings align with European Association of Urology (EAU) recommendations emphasizing serum metabolic profiling in post-transplant management.

The inverse association between serum phosphate and allograft urolithiasis highlights the systemic mineral disturbances typical of post-transplant recovery. Post-transplant hypophosphatemia is common, driven by persistent hyperparathyroidism, elevated FGF-23 levels, and impaired tubular phosphate reabsorption. Low serum phosphate may serve as a surrogate marker for broader mineral renal wasting and altered urinary crystallization dynamics. Future prospective investigations measuring 24-hour urinary phosphate, PTH, FGF-23, and stone composition are warranted to clarify this pathway.

Urinary WBC counts exhibited a powerful exploratory correlation with stone presence. However, pyuria can represent both a predisposing factor (e.g., chronic low-grade inflammation) and a secondary consequence of mechanical urothelial irritation or stasis caused by an existing stone. Furthermore, given the mathematical near-separation observed in statistical modeling, leukocyturia must not be interpreted as a confirmed causal agent or a replacement for microbiological culture data.

Because transplanted kidneys are denervated, urolithiasis may remain clinically silent and be detected during routine imaging follow-up. In this cohort, nearly half of the stones were diagnosed within 6 months after transplantation, although the wide range in time to diagnosis indicates that stone risk persists over the long term. Periodic imaging may therefore be reasonable in selected transplant recipients, particularly when graft dysfunction, urinary symptoms, infection, or metabolic abnormalities are present. However, the present observational study was not designed to define an optimal surveillance interval (8).

Management of allograft lithiasis requires careful consideration of altered pelvic anatomy. Ureteroneocystostomy can complicate retrograde endoscopic approaches, whereas the superficial anterior position of the graft in the iliac fossa may facilitate percutaneous or shockwave modalities (9–13). In our series, individualized interventions successfully preserved graft function without severe procedure-related graft loss.

This study has several limitations. First, it was a retrospective single-center study with only 40 stone events, which limited the complexity of multivariable modeling and does not exclude residual confounding. Second, the study was designed as a risk-set matched case-control analysis rather than a formal time-to-event study; therefore, the 1.74% value represents an observed cumulative proportion rather than a person-time incidence rate. Third, detailed metabolic, microbiological, and transplant-specific evaluations were performed according to clinical indications in routine practice, resulting in variation in the timing and completeness of these assessments. To preserve comparability, the primary matched analysis focused on routinely available variables with adequate completeness across matched sets. Finally, imaging and post-treatment evaluation reflected real-world clinical practice rather than a standardized research protocol, and the exploratory urinary WBC association may have been influenced by reverse causation.

In conclusion, higher serum urate was consistently associated with *de novo* urolithiasis after kidney transplantation, while lower serum phosphate emerged as an additional metabolic signal. Urinary leukocyturia was associated with stone status in exploratory analysis but should not be interpreted as a surrogate for culture-defined urinary tract infection or as a proven causal factor. These findings support careful metabolic assessment and individualized surveillance in kidney transplant recipients and warrant validation in larger prospective multicenter studies.

## Data Availability

The raw data supporting the conclusions of this article will be made available by the authors, without undue reservation.

## Supplementary material

Supplementary Figure S1 Love plot showing the absolute standardized mean differences (SMDs) of the matching covariates before and after 1:2 chronological risk-set matching. The dashed vertical line indicates |SMD|=0.10, the prespecified threshold for acceptable covariate balance.
